# Supplementary material for: No trends in spring and autumn phenology during the global warming hiatus
Source: Nat Commun. 2019 Jun 3;10:2389. doi: 10.1038/s41467-019-10235-8 (PMC6546754; doi:10.1038/s41467-019-10235-8)
Supplement: Supplementary file 1 — Supplementary Information [file 41467_2019_10235_MOESM1_ESM.pdf]

Supplementary Information

No trends in spring and autumn phenology during the global warming hiatus period

Wang et al.

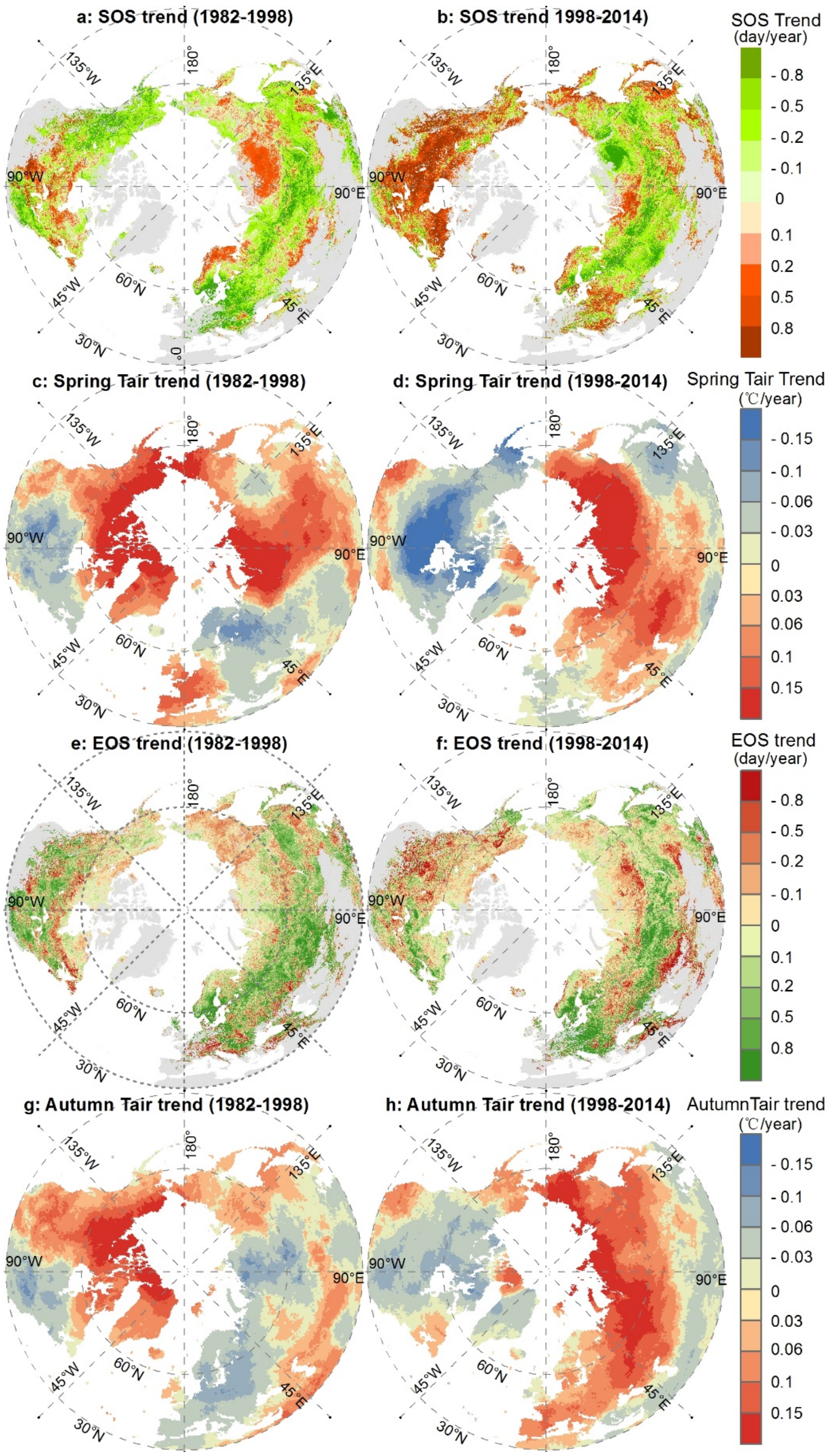

Supplementary Figure 1. Spatial pattern of the remote sensing based phenology trend and temperature trend in the north hemisphere before and during the warming hiatus period. (a) and (b) are the spatial patterns of GIMMS3g based SOS trend before and during the warming hiatus period; (c) and (d) are the spatial patterns of spring temperature trend from CRUTEM4

before and during the warming hiatus period; (e) and (f) are the spatial patterns of GIMMS3g based EOS trend before and during the warming hiatus period; (g) and (h) are the spatial patterns of autumn temperature trend from CRUTEM4 before and during the warming hiatus period.

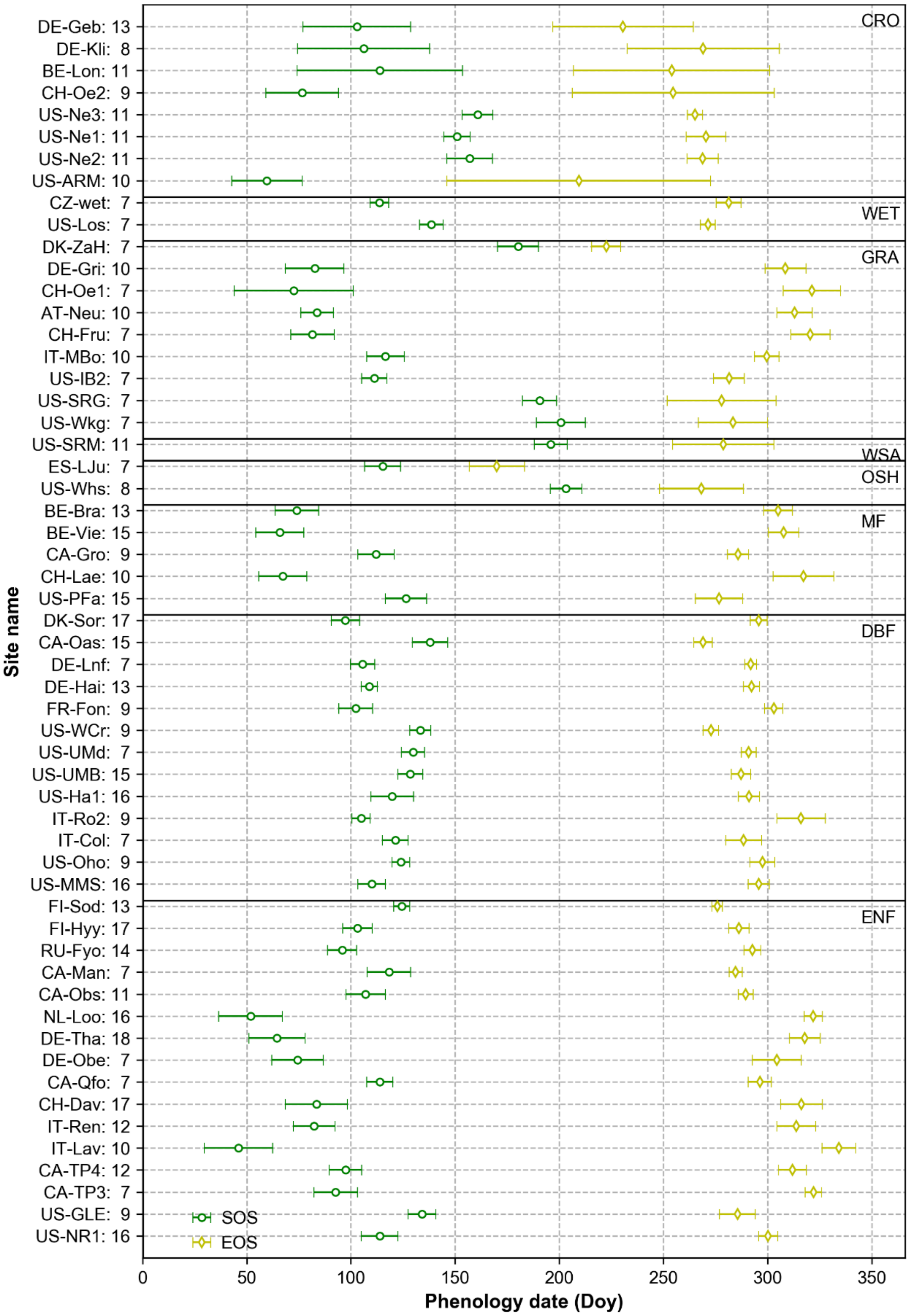

Supplementary Figure 2. Estimated Phenology from FLUXNET GPP data at the sites with at least 7 years of high-quality data. (Tick of Y axis is site ID followed by the number of years with high-quality data to estimate the trend.)

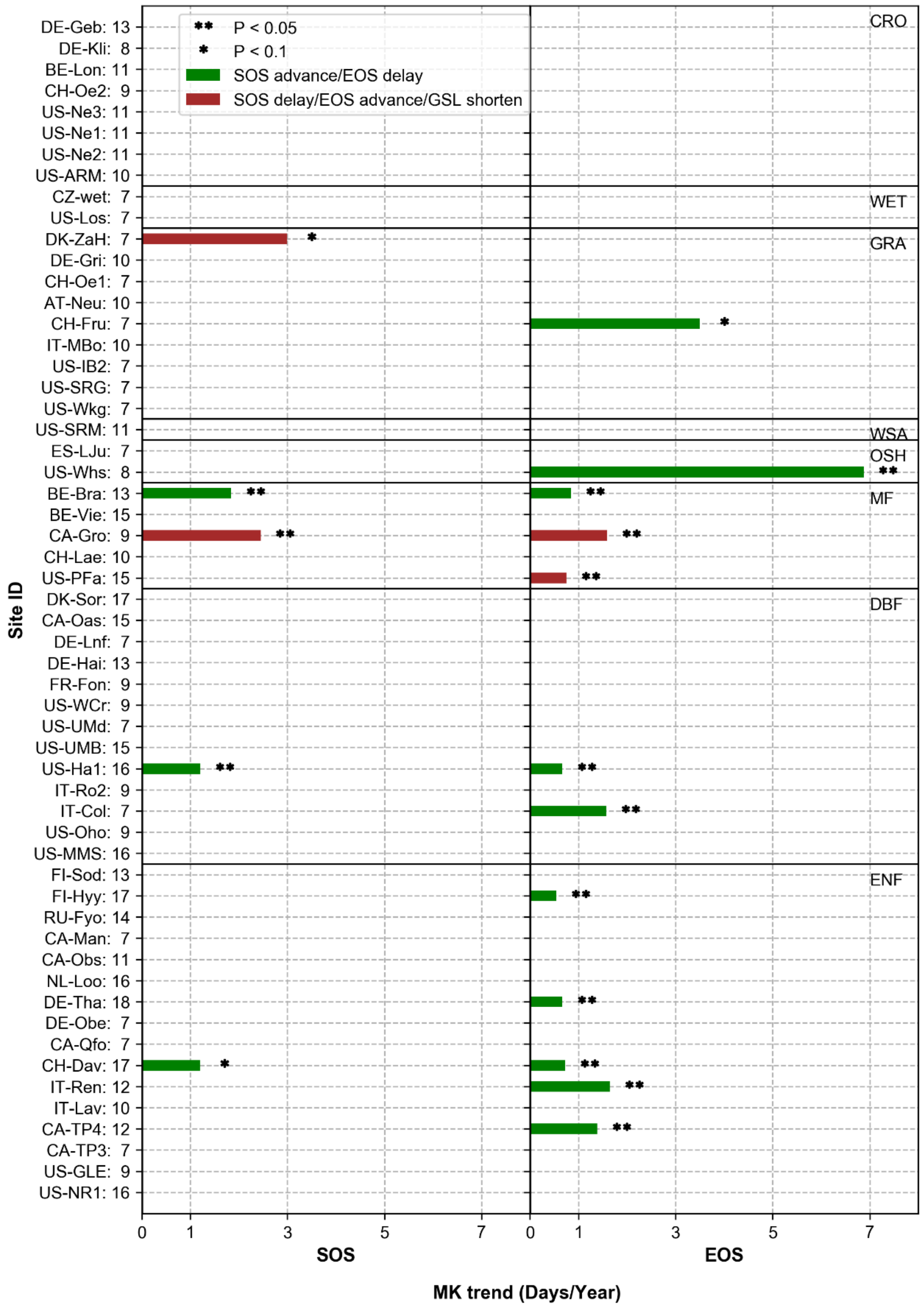

Supplementary Figure 3. Trends of phenological dates (SOS: start of the growing season; EOS: end of the growing season) of the 56 sites. Trends were not plotted for the sites having statistically insignificant trends. The phenological dates were extracted from flux tower GPP data based on the nighttime partitioning method. Tick of Y axis is site ID followed by the

number of years with high quality data to estimate the trend. For each vegetation type, the sites are sorted downward along latitude).

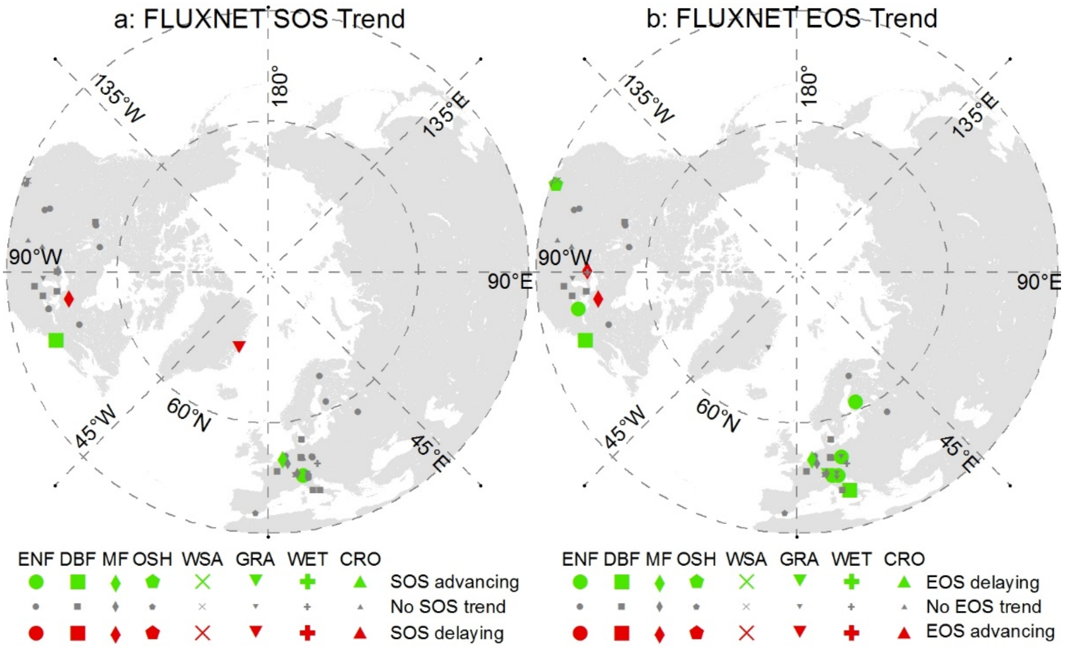

Supplementary Figure 4. The spatial distribution of the FLUXNET sites with significant phenology trends. (a) SOS trends and (b) EOS trends.

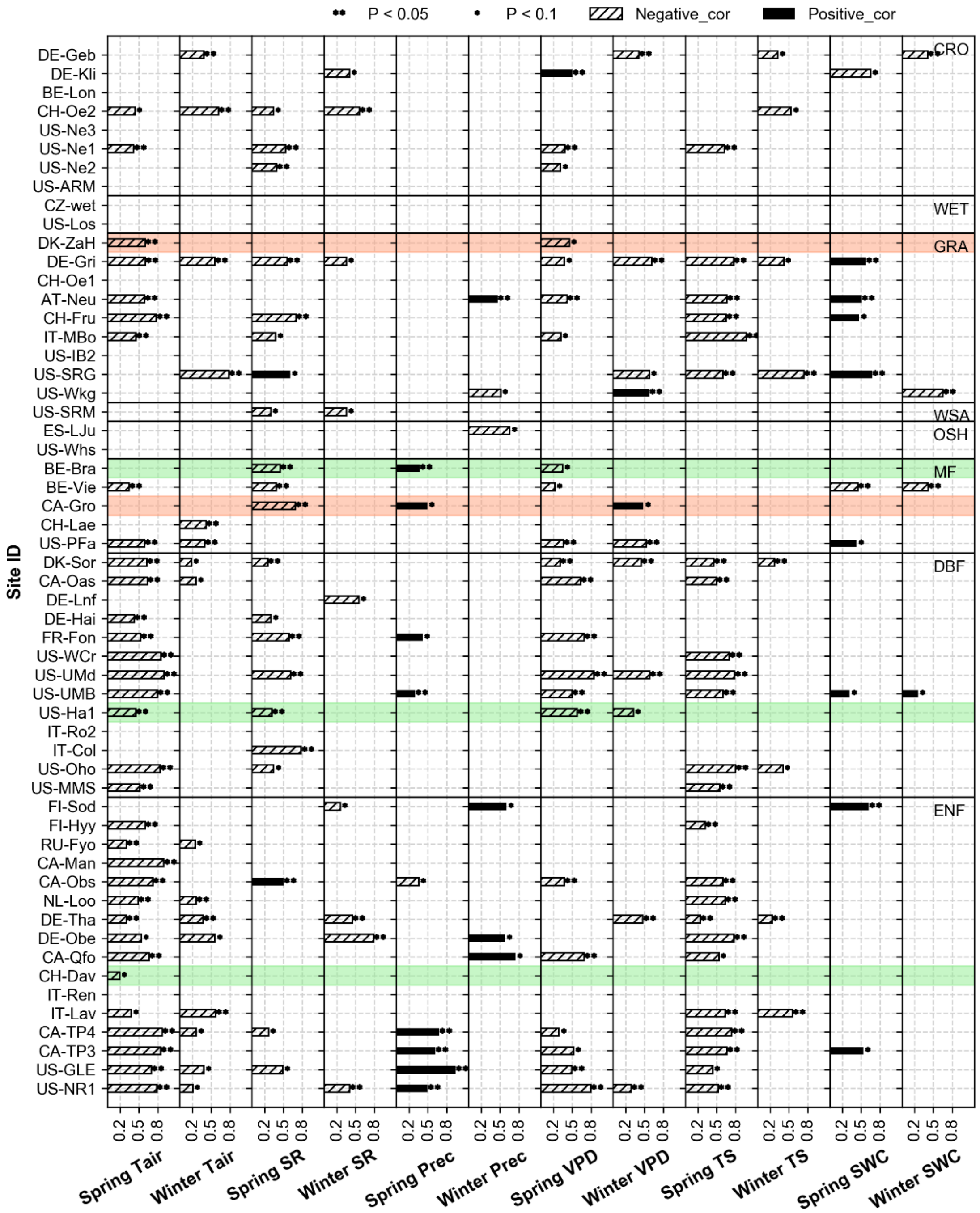

Supplementary Figure 5. Correlation between SOS and each environmental factor in spring and winter. The environmental factors include air temperature (Tair), shortwave radiation (SR), precipitation (Prec), vapor pressure deficit (VPD), soil temperature (TS) and soil water content (SWC) in each month. Light green stands for sites with significant advancing trends in SOS and light yellow for sites with significant delaying trend in SOS. Correlation coefficients were not plotted for the sites with p value greater than 0.1.

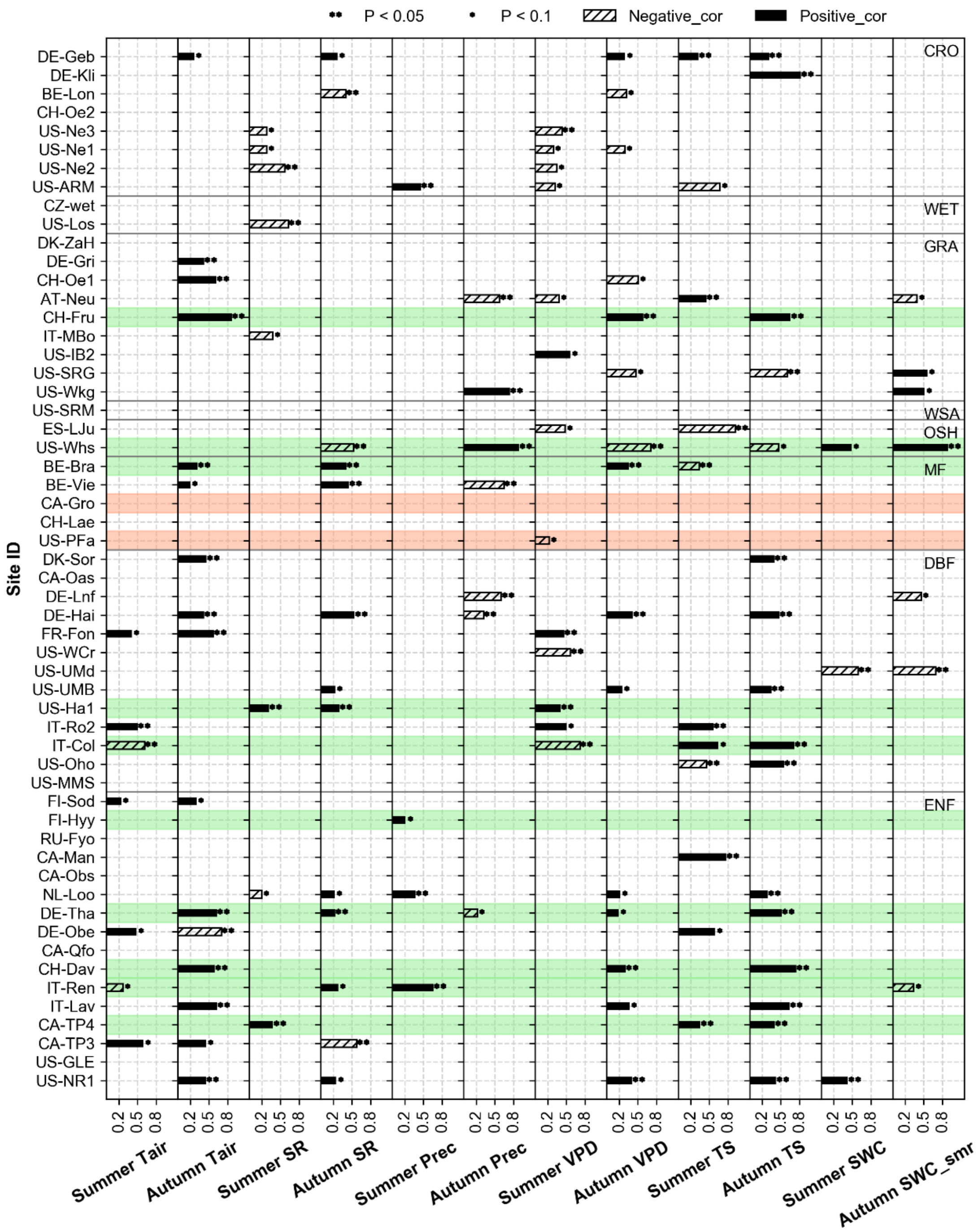

Supplementary Figure 6. Correlation coefficients for the relationships between EOS and environmental factors in summer and autumn, including air temperature (Tair), shortwave radiation (SR), precipitation (Prec), vapor pressure deficit (VPD), soil temperature (TS) and soil water content (SWC). Light green stand for sites with significant delaying EOS trends and light yellow for sites with significant advancing EOS trends. Correlation coefficients were not plotted for the sites with p value greater than 0.1.

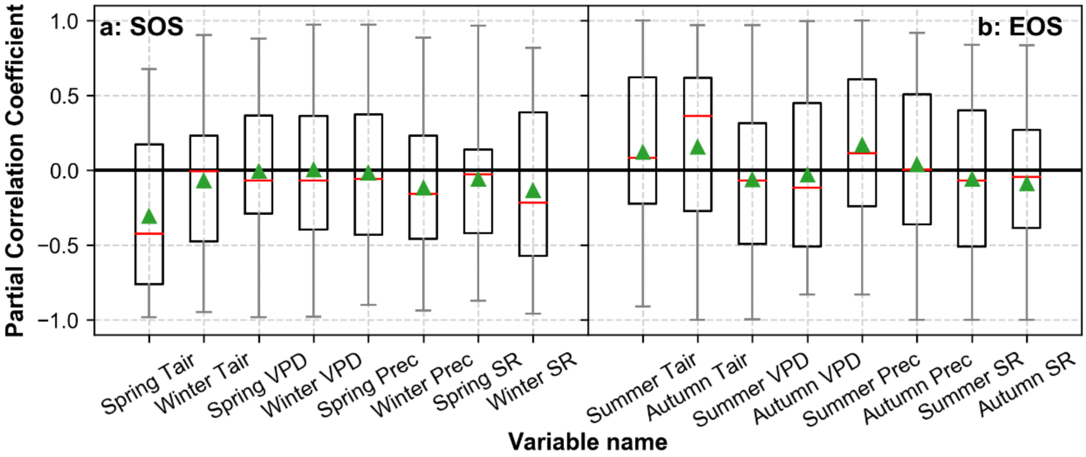

Supplementary Figure 7. Partial correlation coefficient between environmental factors and SOS (or EOS). (a) Temperature (Tair), precipitation (Prec), downward shortwave radiation (SR) and vapor pressure deficit (VPD) in spring and winter was partial correlated with SOS. (b) Temperature, precipitation, downward shortwave radiation and vapor pressure deficit in summer and autumn was partial correlated with EOS. For each box, the central red line is the median, the green triangle is the average, the upper and bottom edge of the box correspond to the 25th and 75th percentiles, the whiskers show the range of the data, and the grey circles are outliers.

IGBP	Site ID	Spring Tair	Winter Tair	Spring SR	Winter SR	Spring Prec	Winter Prec	Spring VPD	Winter VPD	Spring TS	Winter TS	Spring SWC	Winter SWC
CRO	DE-Geb											−2	−1
	DE-Kli												
	BE-Lon												2
	CH-Oe2											2	
	US-Ne3												
	US-Ne1										−1		
	US-Ne2												
	US-ARM												
WET	CZ-wet										−2		
	US-Los												
GRA	DK-ZaH												
	DE-Gri												−2
	CH-Oe1											1	
	AT-Neu					−2				1	2		
	CH-Fru												
	IT-MBo												
	US-IB2		−2										
	US-SRG												
	US-Wkg											2	
WSA	US-SRM			2									
OSH	ES-LJu					2							
	US-Whs												−1
MF	BE-Bra			2				2					
	BE-Vie					−2						1	
	CA-Gro			−1					1				
	CH-Lae											1	2
	US-PFa								−2				
DBF	DK-Sor			2				1				−2	
	CA-Oas												
	DE-Lnf												
	DE-Hai					−1							
	FR-Fon												
	US-WCr						−2		−1			1	
	US-UMd												
	US-UMB											−2	−2
	US-Ha1			2	2			2	1				
	IT-Ro2											−2	
	IT-Col			1					−1	2			
	US-Oho												
	US-MMS												
ENF	FI-Sod			2					−1	2		2	
	FI-Hyy					1	2			1			
	RU-Fyo										2		
	CA-Man				−1		−2				2		
	CA-Obs												
	NL-Loo			2		−2						−2	
	DE-Tha									2		2	2
	DE-Obe												
	CA-Qfo												
	CH-Dav	2			−1	−2		2					
	IT-Ren		−2	1					−2			−2	−2
	IT-Lav												2
	CA-TP4			2						1		−2	
	CA-TP3												−1
	US-GLE									−2	−1		
	US-NR1		−2				2		−1		2		−1

Supplementary Figure 8. Trends of spring air temperature, winter air temperature, spring VPD, winter VPD, spring shortwave radiation, winter shortwave radiation, spring soil temperature, winter soil temperature, spring soil water and winter soil water. Empty cells stand for no trend. Number 1 stands for significant increasing trend with p <0.1. Number 2

stands for significant increasing trend with p <0.05. Number -1 stands for significant decreasing trend with p <0.1. Number -2 stands for significant increasing trend with p <0.05. Light green background in autumn stand for significant SOS advancing trend, light brown background in autumn stand for significant SOS delaying trend.

IGBP	Site ID	Summer Tair	Autumn Tair	Summer SR	Autumn SR	Summer Prec	Autumn Prec	Summer VPD	Autumn VPD	Summer TS	Autumn TS	Summer SWC	Autumn SWC
CRO	DE-Geb											-1	
	DE-Kli												
	BE-Lon												2
	CH-Oe2	-1											1
	US-Ne3					1							
	US-Ne1												
	US-Ne2												
	US-ARM	2			1								
WET	CZ-wet												
	US-Los									1			
GRA	DK-ZaH						2						
	DE-Gri			2						2	2		
	CH-Oe1												
	AT-Neu					-2	-1		-1				-2
	CH-Fru												
	IT-MBo												
	US-IB2												
	US-SRG												
	US-Wkg						1					2	2
WSA	US-SRM						2			2			2
OSH	ES-LJu			-2	-2								
	US-Whs						2				-1		
MF	BE-Bra							2	2	-2			
	BE-Vie		2									1	1
	CA-Gro								2				
	CH-Lae											2	2
	US-PFa				1								
DBF	DK-Sor	1	1										
	CA-Oas						1						
	DE-Lnf												
	DE-Hai												
	FR-Fon						1	-2	-2				
	US-WCr												
	US-UMd												
	US-UMB						2					-2	-2
	US-Ha1			1				2	2				
	IT-Ro2			2	1	-2					1	-2	
	IT-Col				1					2	2		
	US-Oho												
	US-MMS		-1					1					
ENF	FI-Sod									-1		2	
	FI-Hyy				-1					2	2		
	RU-Fyo								-2	-2	2		
	CA-Man	2			-2					-2	2		
	CA-Obs							-1		-1	2		
	NL-Loo						-1			2	2		
	DE-Tha	2	1	2				2		2	1	2	1
	DE-Obe												
	CA-Qfo												
	CH-Dav	2	2			-2		2	1				
	IT-Ren			2	2						-1	-1	-2
	IT-Lav								-1			2	2
	CA-TP4			1							1		
	CA-TP3											-2	
	US-GLE			-1							2		
	US-NR1									-2			-1

Supplementary Figure 9. Trends in summer air temperature, autumn air temperature, summer VPD, autumn VPD, summer shortwave radiation, autumn shortwave radiation, summer soil temperature, autumn soil temperature, summer soil water and autumn soil water. Empty cells stand for no trend. Number 1 stands for significant increasing trend with p <0.1. Number 2

stands for significant increasing trend with $p < 0.05$. Number -1 stands for significant decreasing trend with $p < 0.1$. Number -2 stands for significant increasing trend with $p < 0.05$. Light green background in autumn stand for significant EOS delaying trend, light brown background in autumn stand for significant EOS advancing trend.

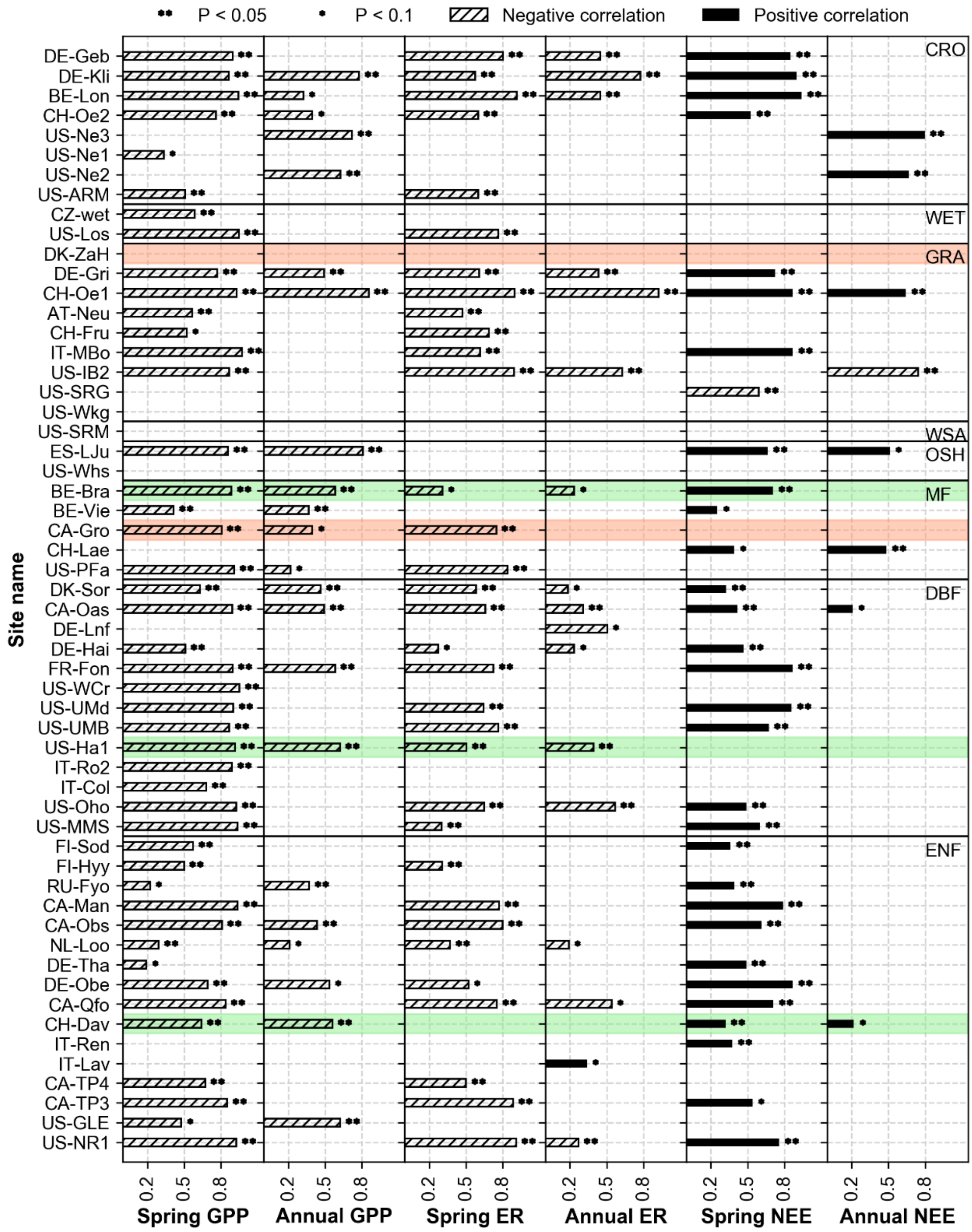

Supplementary Figure 10. Correlation coefficients for the relationships between SOS and carbon fluxes (GPP, ER and NEE) in spring and the whole year. Light green stands for sites with significant SOS advancing trends and light yellow for sites with significant SOS delaying trends.

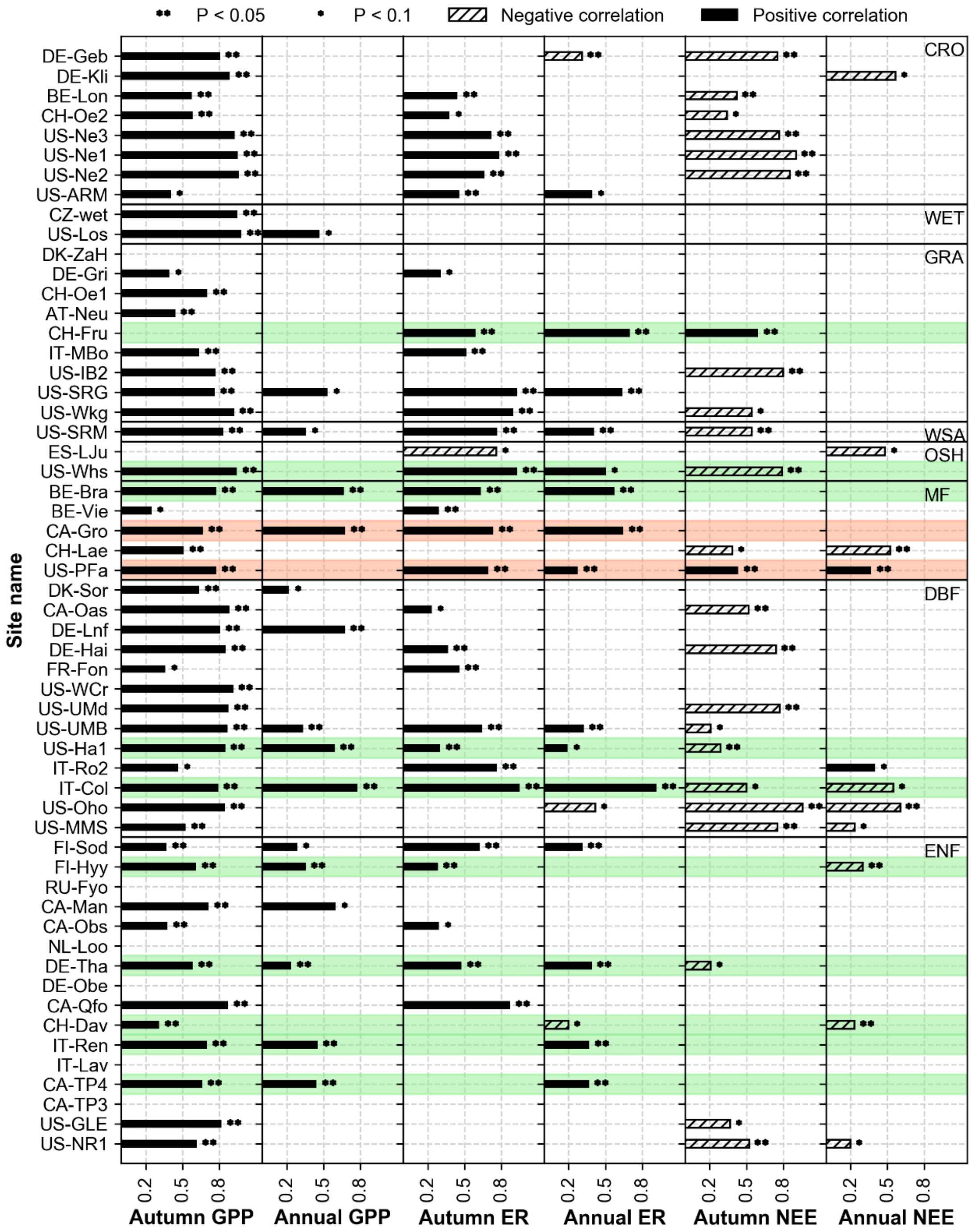

Supplementary Figure 11. Correlation coefficients for the relationships between EOS and carbon fluxes (GPP, ER and NEE) in autumn and the whole year. Light green stands for sites having significant delaying trends in EOS and light yellow for sites having significant advancing trends in EOS.

IGBP	Site ID	Spring GPP	Spring ER	Spring NEE	Autumn GPP	Autumn ER	Autumn NEE
CRO	DE-Geb				2		
	DE-Kli						
	BE-Lon						
	CH-Oe2						
	US-Ne3						
	US-Ne1		1				
	US-Ne2						
	US-ARM						
WET	CZ-wet						
	US-Los						
GRA	DK-ZaH						
	DE-Gri						
	CH-Oe1						
	AT-Neu		1				2
	CH-Fru						2
	IT-MBo						1
	US-IB2						
	US-SRG						
	US-Wkg			2		2	
WSA	US-SRM						
OSH	ES-LJu						
	US-Whs				1	2	
MF	BE-Bra	1		-2	2		
	BE-Vie				2	2	
	CA-Gro	-2	-2		-1	-2	
	CH-Lae			-2	1		-1
	US-PFa	-1			-2	-2	
DBF	DK-Sor			-2	2		-2
	CA-Oas						
	DE-Lnf						
	DE-Hai						
	FR-Fon						
	US-WCr						
	US-UMd						
	US-UMB						
	US-Ha1	1			2	2	
	IT-Ro2						
	IT-Col				1	2	
	US-Oho						
	US-MMS		2				
ENF	FI-Sod						
	FI-Hyy	2	2		2	2	
	RU-Fyo					-2	-2
	CA-Man					-2	
	CA-Obs				1		
	NL-Loo		-2			-1	-1
	DE-Tha		2		2	2	
	DE-Obe						
	CA-Qfo						
	CH-Dav		-2	-2		-2	-2
	IT-Ren	2	2	-1	2	2	
	IT-Lav		-2	-1			
	CA-TP4				2	2	
	CA-TP3				2		-1
	US-GLE						
	US-NR1						

Supplementary Figure 12. trends in GPP, NEE and ER in spring and autumn at each site (light green background in spring stand for significant SOS advancing trend; light brown background in spring stand for significant SOS delaying trend; light green background in autumn stand for significant EOS delaying trend; light brown background in autumn stand for significant EOS advancing trend. Number 1 stands for significant increasing trend with p <0.1. Number 2 stands for significant increasing trend with p <0.05. Number -1 stands for

significant decreasing trend with $p < 0.1$. Number -2 stands for significant decreasing trend with $p < 0.05$.)

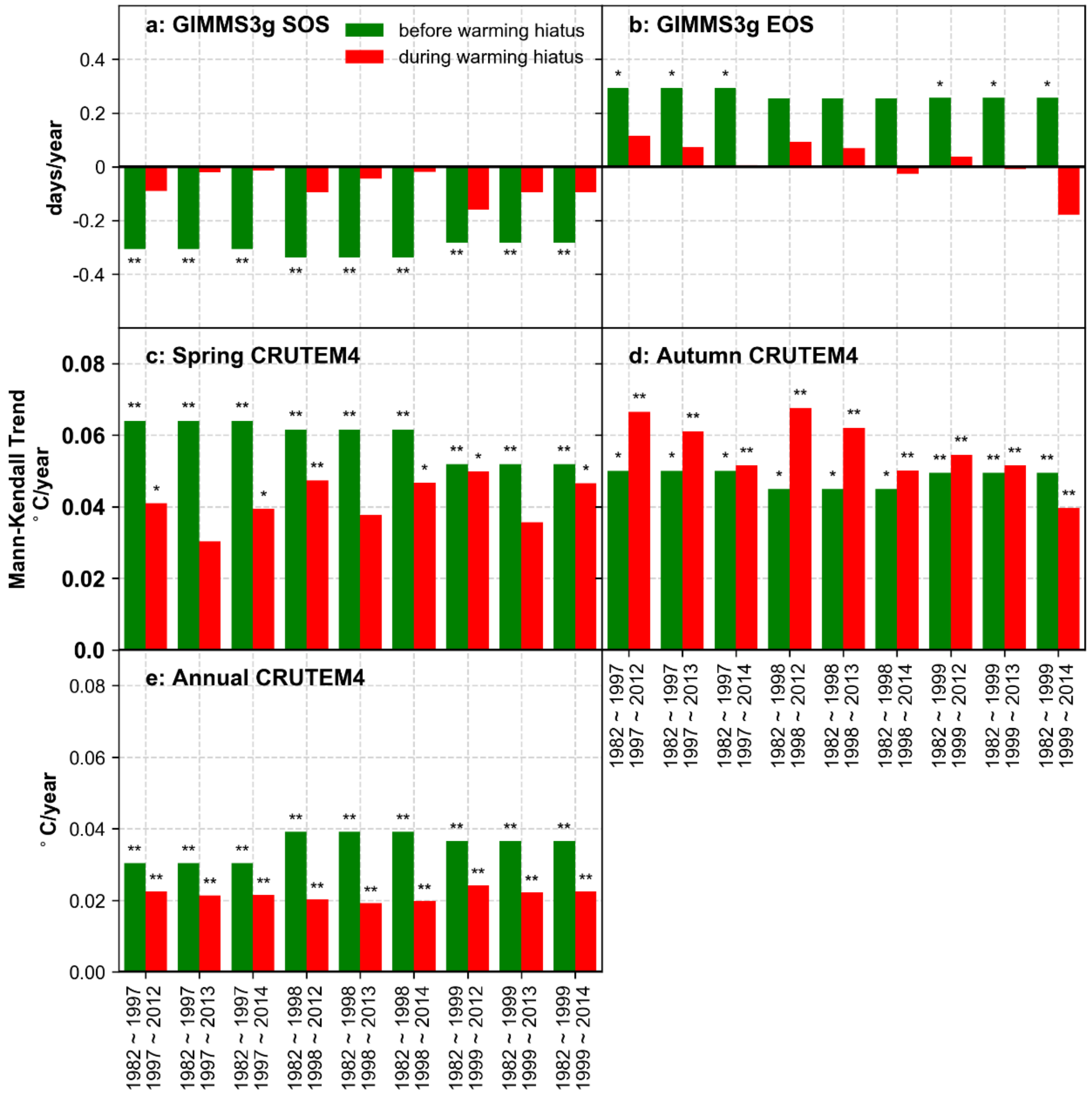

Supplementary Figure 13. Sensitivity test of the trends to the start and end years for the warming hiatus period. (a) Sensitivity test for SOS trends; (b) Sensitivity test for EOS trends; (c) Sensitivity test for spring temperature trends; (b) Sensitivity test for autumn temperature trends; (b) Sensitivity test for annual temperature trends. (* stand for $p < 0.1$, ** stand for $p < 0.05$)

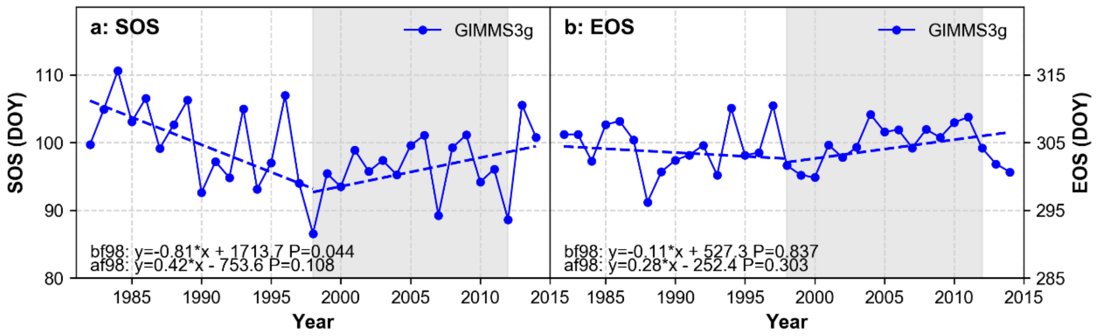

Supplementary Figure 14. GIMMS NDVI3g based SOS (or EOS) trends at the FLUXNET sites. (a) The trend of SOS estimated from GIMMS3g NDVI dataset at the FLUXNET sites. (b) The trend of EOS estimated from GIMMS3g NDVI dataset at the FLUXNET sites. The light grey background indicates the warming hiatus period (1998-2012). (SOS: start of growing season; EOS: end of growing season)

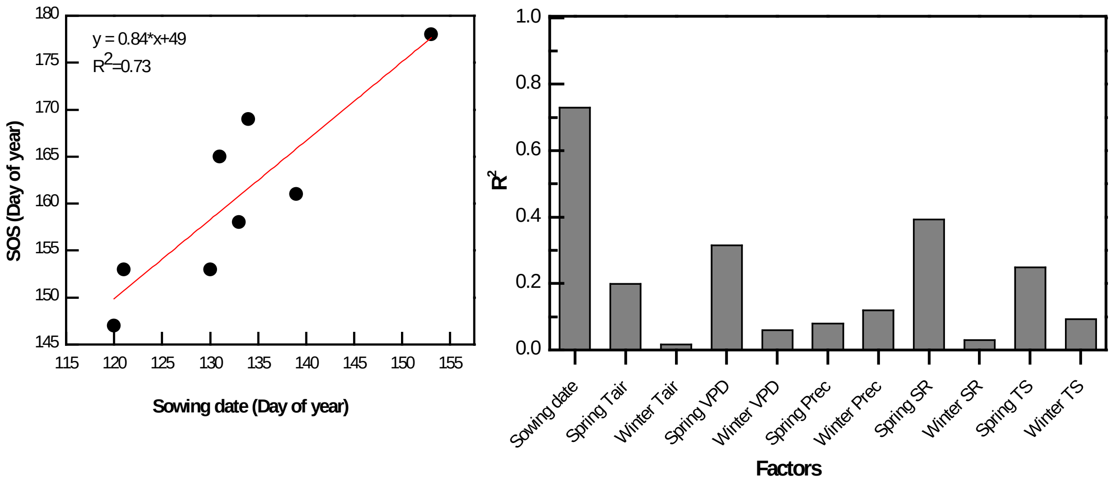

Supplementary Figure 15. The relationships of SOS with climatic factors and sowing date at US-Ne2, a cropland site. The left graph is the scatter plot between the sowing date and SOS. The right graph is a comparison of the coefficient of determination (R^2) between SOS and different factors.

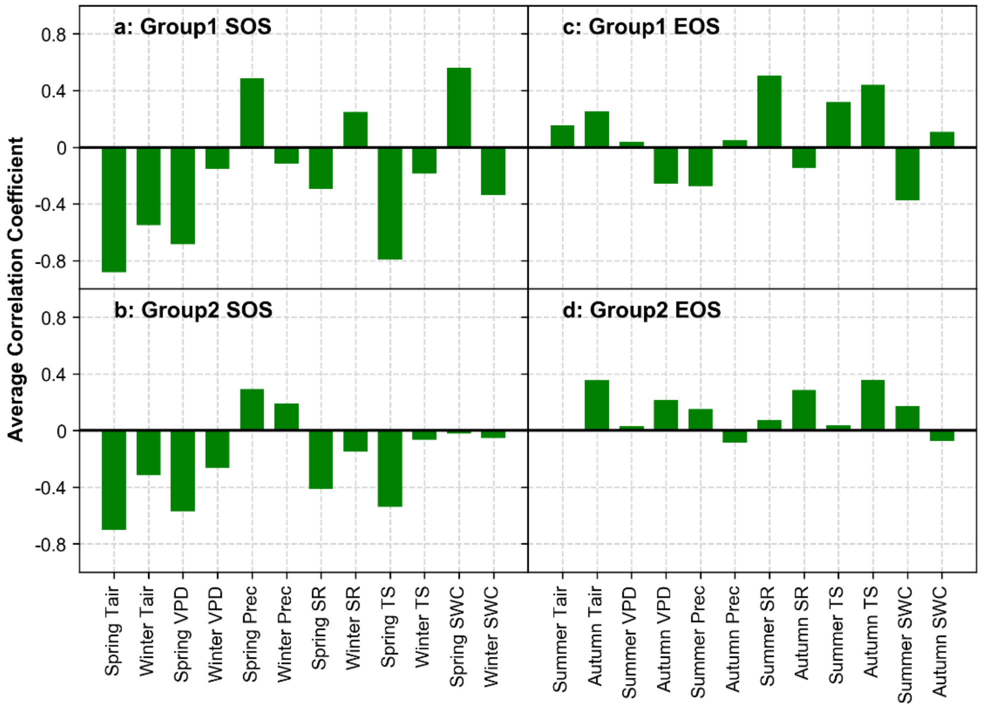

Supplementary Figure 16. Average correlation coefficient between phenology and environmental factors: average correlation coefficient between environmental factors in spring and winter and SOS in group 1 (a) and group 2 (b); average correlation coefficient between environmental factors in spring and winter and EOS in group 1 (c) and group 2 (d).

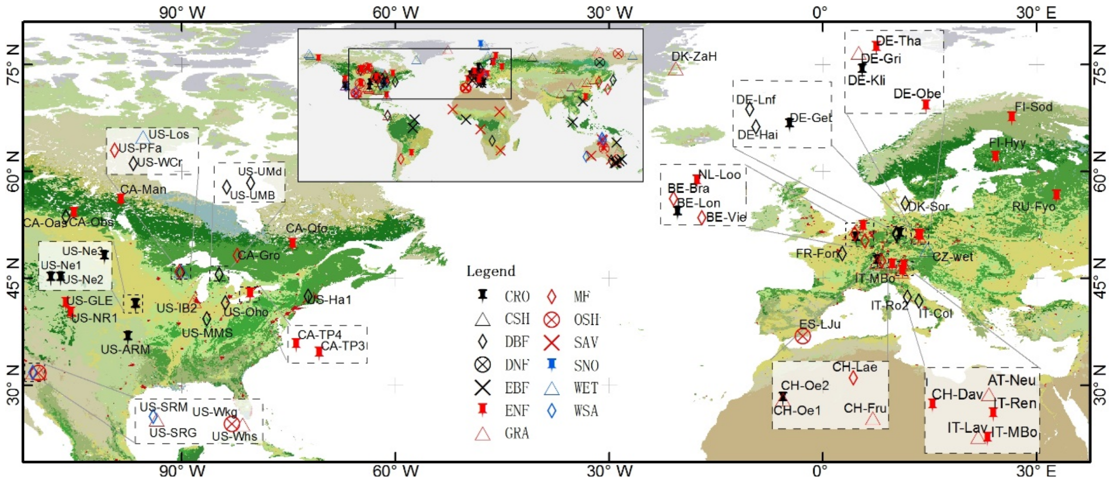

Supplementary Figure 17. The location and distribution of the 56 FLUXNET sites used in this study. Site ID is noted for each site in the map. The inset map shows the location of the 212 sites included in the FLUXNET database. The base map is the MODIS land-cover map in 2010, and the land cover types are as follows: cropland (CRO), closed shrubland (CSH), deciduous broadleaf forest (DBF), deciduous needleleaf forest (DNF), evergreen broadleaf forest (EBF), evergreen needleleaf forest (ENF), grassland (GRA), mixed forest (MF), open shrubland (OSH), savannas (SAV), SNO snow (SNO), wetland (WET), and woody savannas (WSA).

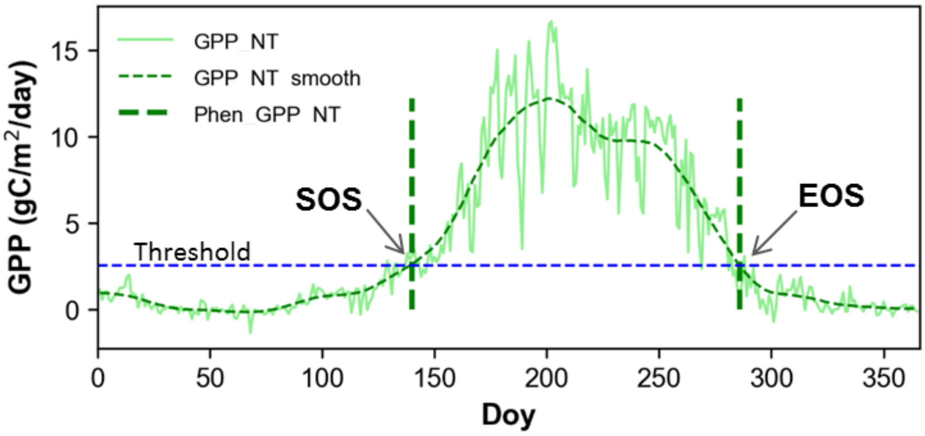

Supplementary Figure 18. Diagram illustrating the extraction of the start of the growing season (SOS) and end of the growing season (EOS) from the daily GPP curve. The GPP data based on the nighttime partitioning method are used in this diagram (light green line). The smoothed GPP data are used for phenology extraction (dashed green curve). The blue dashed line stands for the GPP threshold for extracting the phenological dates.

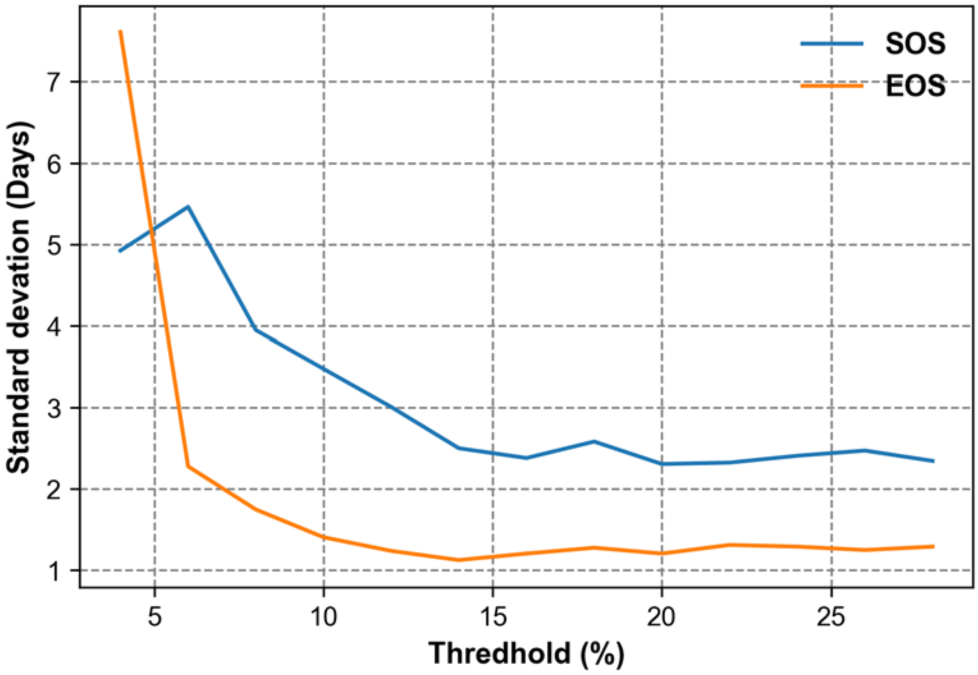

Supplementary Figure 19. Standard deviation of SOS and EOS estimated with the GPP threshold values for US-UMB.

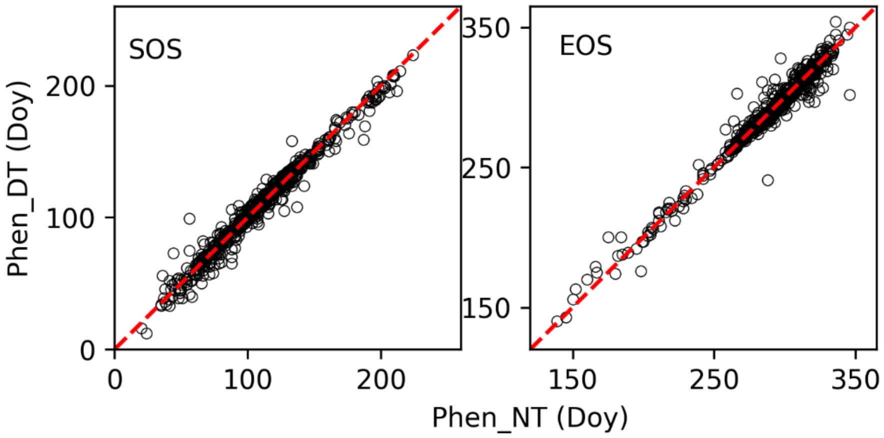

Supplementary Figure 20. Comparison of phenology extracted from GPP partitioned with daytime light use efficiency approach (DT) and nighttime respiration extrapolation approach (NT).

Supplementary Tables

Supplementary Table 1. Average values of correlation coefficient between SOS and environment factors (average for all sites, average for sites of each vegetation type, average for sites that SOS has significant change trends or not).

Average	Number	TA_spr	TA_wit	VPD_spr	VPD_wit	P_spr	P_wit	SW_IN_spr	SW_IN_wit	TS_spr	TS_wit
ALL sites	56	**-0.51**	-0.27	-0.41	-0.21	0.14	0.01	-0.30	-0.05	-0.47	-0.12
Significant sites	5	-0.55	0.10	**-0.56**	-0.17	0.19	0.06	-0.34	-0.17	-0.27	0.27
Non-significant sites	51	**-0.59**	-0.35	-0.46	-0.20	0.25	0.03	-0.35	-0.04	-0.53	-0.17

Supplementary Table 2. Average values of correlation coefficient between EOS and environment factors (average for all sites, average for sites of each vegetation type, average for sites that EOS has significant change trends or not).

Average	Number	TA_smr	TA_aut	VPD_smr	VPD_aut	P_smr	P_aut	SW_IN_smr	SW_IN_aut	TS_smr	TS_aut
ALL sites	56	-0.04	**0.29**	-0.07	0.01	0.03	0.02	0.02	0.06	-0.01	**0.29**
Significant sites	12	-0.11	**0.29**	-0.09	0.00	-0.01	0.02	0.08	0.21	-0.01	0.28
Non-significant sites	44	-0.01	**0.27**	-0.06	0.01	0.03	0.02	0.01	0.02	0.00	0.23

Supplementary Table 3. Average values of correlation coefficient between SOS and carbon fluxes (average for all sites, average for sites of each vegetation type, average for sites that SOS has significant change trends or not).

Average	Number	NEE_spr	NEE_yr	GPP_spr	GPP_yr	RECO_spr	RECO_yr
ALL sites	56	0.50	0.16	**-0.73**	-0.43	-0.53	-0.35
Significant sites	5	0.38	0.13	**-0.72**	-0.59	-0.41	-0.29
Non-significant sites	51	0.53	0.08	**-0.74**	-0.41	-0.54	-0.35

Supplementary Table 4. Average values of correlation coefficient between EOS and carbon fluxes (average for all sites, average for sites of each vegetation type, average for sites that EOS has significant change trends or not).

Average	Number	NEE_aut	NEE_yr	GPP_aut	GPP_yr	RECO_aut	RECO_yr
ALL sites	56	-0.32	-0.17	0.76	0.36	0.55	0.25
Significant sites	12	-0.12	-0.13	0.81	0.63	0.64	0.57
Non-significant sites	44	-0.33	-0.17	0.65	0.27	0.45	0.15

Supplementary Notes

Supplementary Note 1 Comparing trends in environmental variables with SOS (or EOS) trends

To further explore the environmental controls of the phenology trends, we calculated the Mann-Kendall trend for all the meteorological variables for each site (Supplementary Figure 8 and Supplementary Figure 9). We found that for the sites with significant SOS (or EOS) trends, at least one of the significantly correlated meteorological variables had a significant trend. The sites were divided into two groups to conduct the analysis: SOS (or EOS) with a significant trend and SOS (or EOS) without a significant trend.

For the five sites with significant SOS trends, spring air temperature, spring VPD, spring soil temperature and spring shortwave radiation had higher correlation with SOS than did other factors. For all five sites that SOS significantly changed except DK-ZaH, at least one of the environmental factors that were correlated with SOS had a significant trend. For example, SOS at the CH-Dav site was significantly correlated with spring air temperature (Supplementary Figure 5), and spring air temperature had a significant increasing trend (Supplementary Figure 8); SOS at the US-Ha1 site was significantly correlated with spring air temperature, spring shortwave radiation, spring VPD and winter VPD (Supplementary Figure 5), and spring shortwave radiation, spring VPD and winter VPD had significant increasing trends (Supplementary Figure 8).

For the 51 sites without SOS trends, SOS was significantly correlated with spring air temperature for most of these sites (Supplementary Figure 5). However, spring air temperature did not have a significant trend for any of these sites (Supplementary Figure 8). The 51 sites can be divided into four groups. For the first group of sites, some environmental factors were significantly correlated with SOS, but none of these factors had significant trends; these sites include CA-TP3, IT-Lav, CA-Qfo, DE-Obe, NL-Loo, CA-Man, RU-Fyo, CA-Obs, US-MMS, US-Oho, US-UMd, US-WCr, FR-Fon, DE-Hai, DE-Lnf, CA-Oas, CH-Lae, ES-LJu, US-Wkg, US-SRG, IT-MBo, CH-Fru, DE-Gri, US-Ne2, US-Ne1, CH-Oe2 and DK-Kli (Supplementary Figure 5 and Supplementary Figure 8). For the second group of sites (US-NR1, US-GLE, CA-TP4, FI-Hyy, US-UMB, DK-Sor and BE-Vie), several factors were significantly correlated with SOS, but the factors with higher correlation coefficient had

no significant trends (Supplementary Figure 5 and Supplementary Figure 8). Take US-NR1 as an example, SOS had higher correlation with spring air temperature, spring VPD, spring soil temperature and spring precipitation than with winter air temperature and winter VPD (Supplementary Figure 5); spring air temperature, spring VPD, spring soil temperature and spring precipitation had no significant trends although winter air temperature and winter VPD had significant trends (Supplementary Figure 8). For the third group (e.g., IT-Ren, IT-Ro2, US-Whs, US-IB2, CH-Oe1, CZ-Wet and BE-Lon), SOS was not significantly correlated with any environmental factors analyzed; some environmental factors had significant trends (Supplementary Figure 5 and Supplementary Figure 8). For the forth group of sites (US-Los, US-ARM, US-Ne3), none of the environmental factors was correlated with SOS, and no significant trend was found for any of these variables (Supplementary Figure 5 and Supplementary Figure 8). There were also some exceptions such as DE-Tha, FI-Sod, IT-Col, US-PFa, US-SRM, AT-Neu, DE-Geb (Supplementary Figure 5 and Supplementary Figure 8) having significant trends for the environmental factors which were correlated with SOS but having no trends in SOS.

Similarly, we examined the relationship between EOS and each environmental factor in summer and autumn for each site (Supplementary Figure 6). The sites that showed significant EOS trends are highlighted using different backgrounds with light green for sites with significant delaying trends and light yellow for those with significant advancing trends. We first analyzed the sites that EOS significantly changed. In the 12 sites that EOS significantly changed, we found significant trend for the meteorological variable that was significantly correlated with EOS except 4 sites. For example, at the CA-PT4 site (ENF), EOS was positively correlated with summer short wave radiation, summer soil temperature and autumn soil temperature (Supplementary Figure 6); summer short wave radiation and autumn soil temperature had significant increasing trends (Supplementary Figure 9).

The sites without significant EOS trends could be divided into four groups. For the first group of sites (US-GLE, CA-Obs, RU-Fyo, US-MMS, CA-Oas, CH-Lae, US-SRM, DK-ZaH and CH-Oe2), EOS was not highly correlated with any environmental factor, but some environmental factors had significant trends (Supplementary Figure 6 and Supplementary Figure 9). For the second group of sites (CA-Qfo, and CZ-wet), no environmental factor was

significantly correlated with EOS and no significant trends were found for these variables (Supplementary Figure 6 and Supplementary Figure 9). Significant trends were found for the environmental factors that were significantly correlated with EOS for the third group of sites (NL-Loo, CA-Man, FR-Fon, DK-Sor, US-Wkg and AT-Neu) (Supplementary Figure 6 and Supplementary Figure 9). For the remaining 27 sites, some environmental factors in summer or autumn were significantly correlated with EOS and also had significant trends (Supplementary Figure 6 and Supplementary Figure 9).

Supplementary Note 2 Phenology impact on carbon fluxes

To further explore the impacts of phenology trends on carbon fluxes, we examined the sites with significant SOS trends first. For the five sites with significant SOS trends, spring GPP was negatively correlated with SOS at CH-Dav (ENF), US-Ha1 (DBF), CA-Gro (MF) and BE-Bra (MF) (Supplementary Figure 10); the relationship between spring GPP and SOS was not analyzed for DK-ZaH due to the lack of high-quality flux data. Spring GPP had significant increasing trends at US-Ha1 and BE-Bra where SOS significantly advanced, while spring GPP had a significant decreasing trend at CA-Gro where SOS significantly delayed (Supplementary Figure 10 and 12). Spring ecosystem respiration (ER) was also negatively correlated with SOS at US-Ha1, CA-Gro and BE-Bra (Supplementary Figure 10). Spring ER had a significant decreasing trend at CA-Gro where SOS delayed. Spring NEE was positively correlated with SOS at CH-Dav and BE-Bra where SOS advanced, and significant decreasing NEE trends were found at both sites.

We then examined the impacts of phenology trends on carbon fluxes for the 51 sites without significant SOS trends. Carbon fluxes were correlated with SOS and also had significant trends at 11 sites: IT-Lav, IT-Ren, DE-Tha, NL-Loo, FI-Hyy, US-MMS, DK-Sor, US-PFa, CH-Lae, AT-Neu and US-Ne3 (Supplementary Figure 10 and Supplementary Figure 12). At US-Wkg, carbon fluxes were not correlated with SOS but had significant trends (Supplementary Figure 10 and Supplementary Figure 12). Carbon fluxes were not correlated with SOS and also had no trends at 2 sites: US-Whs and US-SRM (Supplementary Figure 10 and Supplementary Figure 12). For the remaining 36 sites, carbon fluxes were correlated with SOS and had no significant trends (Supplementary Figure 10 and Supplementary Figure 12).

We also examined the relationships between EOS and autumn carbon fluxes (Supplementary Figure 11). We first analyzed the EOS impacts on carbon fluxes at the 12 sites with significant EOS trends. Autumn GPP was positively correlated with EOS at all these sites except CH-Fru (Supplementary Figure 11). Autumn GPP significantly increased for eight sites and decreased for two sites (Supplementary Figure 12). Autumn ecosystem respiration was positively correlated with EOS at ten of the 12 sites (Supplementary Figure 11). Among these ten sites, five had delaying EOS trends and increasing autumn ER trends, and two had advancing EOS trends and had decreasing autumn ER trends (Supplementary Figure 12). Autumn NEE was significantly correlated with EOS at six sites (Supplementary Figure 11), one of which had a significant decreasing trend in autumn NEE (Supplementary Figure 12).

We then analyzed the EOS impacts on carbon fluxes at the 44 sites without EOS trends (Supplementary Figure 11 and Supplementary Figure 12). One site (DK-ZaH) were not included in this analysis because of the low quality of autumn carbon fluxes. Carbon fluxes were significantly correlated with EOS and also had significant trends at CA-Obs, CA-Man, DK-Sor, CH-Lae, BE-Vie, US-Wkg, IT-MBo, AT-Neu and DE-Geb. At CA-TP3, NL-Loo and RU-Fyo, carbon fluxes were not correlated with EOS but had significant trends. Carbon fluxes were not correlated with EOS and had no trends at IT-Lav. For the remaining 31 sites, carbon fluxes were significantly correlated with EOS and had no significant trends.

Supplementary References

1. Moureaux C, Debacq A, Bodson B, Heinesch B, Aubinet M. Annual net ecosystem carbon exchange by a sugar beet crop. *Agricultural and Forest Meteorology* **139**, 25-39 (2006).
2. Kutsch W, et al. The net biome production of full crop rotations in Europe. *Agriculture, ecosystems & environment* **139**, 336-345 (2010).
3. Anthoni P, et al. Forest and agricultural land‐use‐dependent CO2 exchange in Thuringia, Germany. *Global Change Biol* **10**, 2005-2019 (2004).
4. Kutsch WL, et al. The net biome production of full crop rotations in Europe. *Agriculture, Ecosystems & Environment* **139**, 336-345 (2010).
5. Fischer ML, Billesbach DP, Berry JA, Riley WJ, Torn MS. Spatiotemporal Variations in Growing Season Exchanges of CO2, H2O, and Sensible Heat in Agricultural Fields of the Southern Great Plains. *Earth Interactions* **11**, 1-21 (2007).
6. Suyker AE, Verma SB, Burba GG, Arkebauer TJ. Gross primary production and ecosystem respiration of irrigated maize and irrigated soybean during a growing season. *Agricultural and*

Forest Meteorology **131**, 180-190 (2005).

7. Barr AG, et al. Comparing the carbon budgets of boreal and temperate deciduous forest stands. *Can J Forest Res* **32**, 813-822 (2002).
8. Pilegaard K, Mikkelsen TN, Beier C, Jensen NO, Ambus P, Ro-Poulsen H. Field measurements of atmosphere-biosphere interactions in a Danish beech forest. *Boreal Environment Research* **8**, 315-334 (2003).
9. Delpierre N, Berveiller D, Granda E, Dufrêne E. Wood phenology, not carbon input, controls the interannual variability of wood growth in a temperate oak forest. *New Phytol* **210**, 459-470 (2016).
10. VALENTINI R, ANGELIS P, MATTEUCCI G, MONACO R, DORE S, MUCNOZZA GES. Seasonal net carbon dioxide exchange of a beech forest with the atmosphere. *Global Change Biol* **2**, 199-207 (1996).
11. Tedeschi V, Rey A, Manca G, Valentini R, Jarvis PG, Borghetti M. Soil respiration in a Mediterranean oak forest at different developmental stages after coppicing. *Global Change Biol* **12**, 110-121 (2006). 12. Barford CC, et al. Factors Controlling Long- and Short-Term Sequestration of Atmospheric CO₂ in a Mid-latitude Forest. Science 294, 1688-1691 (2001).
13. Oliphant AJ, et al. Heat storage and energy balance fluxes for a temperate deciduous forest. *Agricultural and Forest Meteorology* **126**, 185-201 (2004).
14. DeForest JL, Noormets A, McNulty SG, Sun G, Tenney G, Chen J. Phenophases alter the soil respiration–temperature relationship in an oak-dominated forest. *International journal of biometeorology* **51**, 135-144 (2006).
15. Curtis PS, et al. Biometric and eddy-covariance based estimates of annual carbon storage in five eastern North American deciduous forests. *Agricultural and Forest Meteorology* **113**, 3-19 (2002).
16. Nave LE, et al. Disturbance and the resilience of coupled carbon and nitrogen cycling in a north temperate forest. *Journal of Geophysical Research: Biogeosciences* **116**, (2011).
17. Cook BD, et al. Carbon exchange and venting anomalies in an upland deciduous forest in northern Wisconsin, USA. *Agricultural and Forest Meteorology* **126**, 271-295 (2004).
18. Hirsch AI, Trumbore SE, Goulden ML. The surface CO2 gradient and pore-space storage flux in a high-porosity litter layer. *Tellus B: Chemical and Physical Meteorology* **56**, 312-321 (2004).
19. Bond-Lamberty B, Wang C, Gower ST. A global relationship between the heterotrophic and autotrophic components of soil respiration? *Global Change Biol* **10**, 1756-1766 (2004).
20. Bergeron O, et al. Comparison of carbon dioxide fluxes over three boreal black spruce forests in Canada. *Global Change Biol* **13**, 89-107 (2007).
21. Arain MA, Restrepo-Coupe N. Net ecosystem production in a temperate pine plantation in southeastern Canada. *Agricultural and Forest Meteorology* **128**, 223-241 (2005).
22. Arain MA, Yuan F, Black TA. Soil–plant nitrogen cycling modulated carbon exchanges in a western temperate conifer forest in Canada. *Agricultural and Forest Meteorology* **140**, 171-192 (2006).
23. Zweifel R, Eugster W, Etzold S, Dobbertin M, Buchmann N, Häsler R. Link between continuous stem radius changes and net ecosystem productivity of a subalpine Norway spruce forest in the Swiss Alps. *New Phytol* **187**, 819-830 (2010).
24. GrüNwald T, Bernhofer C. A decade of carbon, water and energy flux measurements of an old spruce forest at the Anchor Station Tharandt. *Tellus B: Chemical and Physical Meteorology* **59**, 387-396 (2007).

25. Suni T, et al. Long-term measurements of surface fluxes above a Scots pine forest in Hyytiala, southern Finland, 1996-2001. *Boreal Environment Research* **8**, 287-302 (2003).
26. Tanja S, et al. Air temperature triggers the recovery of evergreen boreal forest photosynthesis in spring. *Global Change Biol* **9**, 1410-1426 (2003).
27. Fiora A, Cescatti A. Diurnal and seasonal variability in radial distribution of sap flux density: implications for estimating stand transpiration. *Tree Physiol* **26**, 1217-1225 (2006).
28. Feigenwinter C, Montagnani L, Aubinet M. Plot-scale vertical and horizontal transport of CO2 modified by a persistent slope wind system in and above an alpine forest. *Agricultural and Forest Meteorology* **150**, 665-673 (2010).
29. Dolman A, Moors E, Elbers J. The carbon uptake of a mid latitude pine forest growing on sandy soil. *Agricultural and Forest Meteorology* **111**, 157-170 (2002).
30. Kurbatova J, Li C, Varlagin A, Xiao X, Vygodskaya N. Modeling carbon dynamics in two adjacent spruce forests with different soil conditions in Russia. *Biogeosciences* **5**, 969-980 (2008).
31. Bradford JB, Birdsey RA, Joyce LA, Ryan MG. Tree age, disturbance history, and carbon stocks and fluxes in subalpine Rocky Mountain forests. *Global Change Biol* **14**, 2882-2897 (2008).
32. Monson R, et al. Carbon sequestration in a high‐elevation, subalpine forest. *Global Change Biology* **8**, 459-478 (2002).
33. Wohlfahrt G, Bahn M, Haslwanter A, Newesely C, Cernusca A. Estimation of daytime ecosystem respiration to determine gross primary production of a mountain meadow. *Agricultural and Forest Meteorology* **130**, 13-25 (2005).
34. Eugster W, Zeeman MJ. Micrometeorological techniques to measure ecosystem-scale greenhouse gas fluxes for model validation and improvement. In: International Congress Series (ed^(eds). Elsevier (2006).
35. Ammann C, Flechard CR, Leifeld J, Neftel A, Fuhrer J. The carbon budget of newly established temperate grassland depends on management intensity. *Agriculture, Ecosystems & Environment* **121**, 5-20 (2007).
36. Prescher A-K, Grünwald T, Bernhofer C. Land use regulates carbon budgets in eastern Germany: From NEE to NBP. *Agricultural and Forest Meteorology* **150**, 1016-1025 (2010).
37. Soegaard H, Nordstroem C. Carbon dioxide exchange in a high-arctic fen estimated by eddy covariance measurements and modelling. *Global Change Biol* **5**, 547-562 (1999).
38. Wohlfahrt G, Sapinsky S, Tappeiner U, Cernusca A. Estimation of plant area index of grasslands from measurements of canopy radiation profiles. *Agricultural and Forest Meteorology* **109**, 1-12 (2001).
39. Jastrow JD. CHANGES IN SOIL AGGREGATION ASSOCIATED WITH TALLGRASS PRAIRIE RESTORATION. *Am J Bot* **74**, 1656-1664 (1987).
40. Scott RL, Biederman JA, Hamerlynck EP, Barron‐Gafford GA. The carbon balance pivot point of southwestern US semiarid ecosystems: Insights from the 21st century drought. *Journal of Geophysical Research: Biogeosciences* **120**, 2612-2624 (2015).
41. Biederman JA, et al. Terrestrial carbon balance in a drier world: the effects of water availability in southwestern North America. *Global Change Biol* **22**, 1867-1879 (2016).
42. Churkina G, et al. Analyzing the Ecosystem Carbon Dynamics of Four European Coniferous Forests Using a Biogeochemistry Model. *Ecosystems* **6**, 0168-0184 (2003).
43. Aubinet M, Chermanne B, Vandenhaute M, Longdoz B, Yernaux M, Laitat E. Long term carbon dioxide exchange above a mixed forest in the Belgian Ardennes. *Agricultural and Forest*

Meteorology **108**, 293-315 (2001).

44. McCaughey JH, Pejam MR, Arain MA, Cameron DA. Carbon dioxide and energy fluxes from a boreal mixedwood forest ecosystem in Ontario, Canada. *Agricultural and Forest Meteorology* **140**, 79-96 (2006).
45. Eugster W, et al. Methodical study of nitrous oxide eddy covariance measurements using quantum cascade laser spectrometery over a Swiss forest. *Biogeosciences* **4**, 927-939 (2007).
46. Davis KJ, et al. The annual cycles of CO2 and H2O exchange over a northern mixed forest as observed from a very tall tower. *Global Change Biology* **9**, 1278-1293 (2003).
47. Serrano-Ortiz P, et al. Variations in daytime net carbon and water exchange in a montane shrubland ecosystem in southeast Spain. *Photosynthetica* **45**, 30-35 (2007).
48. Hamerlynck EP, Scott RL, Sánchez-Cañete EP, Barron-Gafford GA. Nocturnal soil CO2 uptake and its relationship to subsurface soil and ecosystem carbon fluxes in a Chihuahuan Desert shrubland. *Journal of Geophysical Research: Biogeosciences* **118**, 1593-1603 (2013).
49. Dušek J, Čížková H, Stellner S, Czerný R, Květ J. Fluctuating water table affects gross ecosystem production and gross radiation use efficiency in a sedge-grass marsh. *Hydrobiologia* **692**, 57-66 (2012).
50. Scott Denning A, et al. Simulated variations in atmospheric CO2 over a Wisconsin forest using a coupled ecosystem–atmosphere model. *Global Change Biology* **9**, 1241-1250 (2003).
51. Scott RL, Jenerette GD, Potts DL, Huxman TE. Effects of seasonal drought on net carbon dioxide exchange from a woody‐plant‐encroached semiarid grassland. *Journal of Geophysical Research: Biogeosciences* **114**, (2009).
