# Supplementary material for: No trends in spring and autumn phenology during the global warming hiatus
Source: Nat Commun. 2019 Jun 3;10:2389. doi: 10.1038/s41467-019-10235-8 (PMC6546754; doi:10.1038/s41467-019-10235-8)
Supplement: Supplementary file 3 — Description of Additional Supplementary Files [file 41467_2019_10235_MOESM3_ESM.pdf]

Description of Additional Supplementary Files

File Name: Supplementary Data 1

Description: Information on the FLUXNET sites used in this study.
