# Supplementary material for: No trends in spring and autumn phenology during the global warming hiatus
Source: Nat Commun. 2019 Jun 3;10:2389. doi: 10.1038/s41467-019-10235-8 (PMC6546754; doi:10.1038/s41467-019-10235-8)
Supplement: Supplementary file 4 — Supplementary Data 1 [file 41467_2019_10235_MOESM4_ESM.pdf]

Sitename	Site ID	IGBP	lat	lon	ELEV	Start year	End year	Years	Years_QC>0.75	Reference
Lonzee	BE-Lon	CRO	50.5516	4.7461	167	2004	2014	11	11	[1]
Oensingen crop	CH-Oe2	CRO	47.2863	7.7343	452	2004	2014	11	9	[2]
Gebesee	DE-Geb	CRO	51.1001	10.9143	161.5	2001	2014	14	13	[3]
Klingenberg	DE-Kli	CRO	50.8929	13.5225	478	2004	2014	11	8	[4]
ARM Southern Great Plains site-Lamont	US-ARM	CRO	36.6058	-97.4888	314	2003	2012	10	10	[5]
Mead - irrigated continuous maize site	US-Ne1	CRO	41.1651	-96.4766	361	2001	2013	13	11	[6]
Mead - irrigated maize-soybean rotation site	US-Ne2	CRO	41.1649	-96.4701	362	2001	2013	13	11	[6]
Mead - rainfed maize-soybean rotation site	US-Ne3	CRO	41.1797	-96.4397	363	2001	2013	13	11	[6]
Saskatchewan - Western Boreal, Mature Aspen	CA-Oas	DBF	53.6289	-106.1978	530	1996	2010	15	15	[7]
Hainich	DE-Hai	DBF	51.0792	10.4530	430	2000	2012	13	13	[3]
Leinefelde	DE-Lnf	DBF	51.3282	10.3678	451	2002	2012	11	7	[3]
Soroe	DK-Sor	DBF	55.4859	11.6446	40	1996	2014	19	17	[8]
Fontainebleau-Barbeau	FR-Fon	DBF	48.4764	2.7801	103	2005	2014	10	9	[9]
Grignon	IT-Col	DBF	41.8494	13.5881	1560	1996	2014	19	7	[10]
Roccarespampani 2	IT-Ro2	DBF	42.3903	11.9209	160	2002	2012	11	9	[11]
Harvard Forest EMS Tower (HFR1)	US-Ha1	DBF	42.5378	-72.1715	340	1991	2012	22	16	[12]
Morgan Monroe State Forest	US-MMS	DBF	39.3232	-86.4131	275	1999	2014	16	16	[13]
Oak Openings	US-Oho	DBF	41.5545	-83.8438	230	2004	2013	10	9	[14]
Univ. of Mich. Biological Station	US-UMB	DBF	45.5598	-84.7138	234	2000	2014	15	15	[15]
	US-UMd	DBF	45.5625	-84.6975	239	2007	2014	8	7	[16]

Willow Creek	US-WCr	DBF	45.8059	-90.0799	520	1999	2014	16	9	[17]
Manitoba - Northern Old Black Spruce (former BOREAS Northern Study Area)	CA-Man	ENF	55.8796	-98.4808	259	1994	2008	15	7	[18]
Saskatchewan - Western Boreal, Mature Black Spruce	CA-Obs	ENF	53.9872	-105.1178	628.94	1997	2010	14	11	[19]
Quebec - Eastern Boreal, Mature Black Spruce	CA-Qfo	ENF	49.6925	-74.3421	382	2003	2010	8	7	[20]
Ontario - Turkey Point 1974 Plantation White Pine	CA-TP3	ENF	42.7068	-80.3483	184	2002	2014	13	7	[21]
Turkey Point White Pine 1939	CA-TP4	ENF	42.7102	-80.3574	184	2002	2014	13	12	[22]
Davos	CH-Dav	ENF	46.8153	9.8559	1639	1997	2014	18	17	[23]
Oberbärenburg	DE-Obe	ENF	50.7836	13.7196	735	2008	2014	7	7	
Tharandt	DE-Tha	ENF	50.9636	13.5669	380	1996	2014	19	18	[24]
Hyytiala	FI-Hyy	ENF	61.8475	24.2950	181	1996	2014	19	17	[25]
Sodankylä	FI-Sod	ENF	67.3619	26.6378	180	2001	2014	14	13	[26]
Lavarone	IT-Lav	ENF	45.9562	11.2813	1353	2003	2014	12	10	[27]
Renon	IT-Ren	ENF	46.5869	11.4337	1730	1998	2013	16	12	[28]
Loobos	NL-Loo	ENF	52.1666	5.7436	25	1996	2014	19	16	[29]
Fyodorovskoye	RU-Fyo	ENF	56.4615	32.9221	265	1998	2014	17	14	[30]
GLEES	US-GLE	ENF	41.3665	-106.2399	3197	2004	2014	11	9	[31]
Niwot Ridge Forest (LTER NWT1)	US-NR1	ENF	40.0329	-105.5464	3050	1998	2014	17	16	[32]
Neustift	AT-Neu	GRA	47.1167	11.3175	970	2002	2012	11	10	[33]
Früebüel	CH-Fru	GRA	47.1158	8.5378	982	2005	2014	10	7	[34]
Oensingen grassland	CH-Oe1	GRA	47.2858	7.7319	450	2002	2008	7	7	[35]
Grillenburg	DE-Gri	GRA	50.9495	13.5125	385	2004	2014	11	10	[36]
Zackenberg Heath	DK-ZaH	GRA	74.4732	-20.5503	38	2000	2014	15	7	[37]
Monte Bondone	IT-MBo	GRA	46.0147	11.0458	1550	2003	2013	11	10	[38]

Fermi National Accelerator Laboratory- Batavia (Prairie site)	US-IB2	GRA	41.8406	-88.2410	226.5	2004	2011	8	7	[39]
Santa Rita Grassland	US-SRG	GRA	31.7894	-110.8277	1291	2008	2014	7	7	[40]
Walnut Gulch Kendall Grasslands	US-Wkg	GRA	31.7365	-109.9419	1531	2004	2014	11	7	[41]
Brasschaat	BE-Bra	MF	51.3092	4.5206	16	1996	2014	19	13	[42]
Vielsalm	BE-Vie	MF	50.3051	5.9981	493	1996	2014	19	15	[43]
Ontario - Groundhog River, Boreal Mixedwood Forest	CA-Gro	MF	48.2167	-82.1556	340	2003	2014	12	9	[44]
Laegern	CH-Lae	MF	47.4781	8.3650	689	2004	2014	11	10	[45]
Park Falls/WLEF	US-PFa	MF	45.9459	-90.2723	470	1995	2014	20	15	[46]
Llano de los Juanes	ES-LJu	OSH	36.9266	-2.7521	1600	2004	2013	10	7	[47]
Walnut Gulch Lucky Hills Shrub	US-Whs	OSH	31.7438	-110.0522	1370	2007	2014	8	8	[48]
Trebon (CZECHWET)	CZ-wet	WET	49.0247	14.7704	426	2006	2014	9	7	[49]
Lost Creek	US-Los	WET	46.0827	-89.9792	480	2000	2014	15	7	[50]
Santa Rita Mesquite	US-SRM	WSA	31.8214	-110.8661	1120	2004	2014	15	11	[51]
